# The Swelling That Would Not Settle: A Four-Month Journey to Diagnosing a Morel-Lavallée Lesion

**DOI:** 10.7759/cureus.110355

**Published:** 2026-06-06

**Authors:** Imran F Gul, Mohd Shahril Jaapar, Syahril Izwan Alias

**Affiliations:** 1 Orthopaedics, Universiti Putra Malaysia, Serdang, MYS; 2 Foot and Ankle, Orthopaedics and Traumatology, Universiti Putra Malaysia Medical Faculty, Serdang, MYS; 3 Orthopaedics and Traumatology, Hospital Sultan Abdul Aziz Shah, Universiti Putra Malaysia, Serdang, MYS

**Keywords:** chronic swelling, closed degloving injury, morel-lavallee, post-traumatic swelling, thigh swelling

## Abstract

Morel-Lavallée lesions (MLL) are closed degloving injuries caused by shearing forces that separate the skin and tissue from the deep fascia. This creates a space where blood and lymphatic fluid can collect if not found early. MLL often happen after high-energy trauma and may be missed when other injuries seem more urgent. A 35-year-old motorcyclist developed a swelling on his left thigh after an accident. At first, it was thought to be just a bruise. Over the next few months, the swelling grew and became more painful. Ultrasound and CT scans showed a large, multiloculated fluid collection, which matched a chronic MLL. Surgery drained about one liter of fluid through two incisions. After the wound was closed, the patient recovered fully without complications. This case shows that MLL can be missed, especially when the first injury seems minor. If the condition becomes chronic, surgery is often needed because a fibrous capsule forms around the fluid. If swelling lasts after an injury, clinicians should consider MLL. Early imaging and quick treatment can lower the risk of complications and help patients recover better.

## Introduction

Morel-Lavallée lesion (MLL) is a closed soft-tissue injury caused by strong shearing forces, first described by Morel-Lavallée in 1863. MLL usually occurs in areas where the skin is highly mobile and richly vascularized, such as the lateral thigh, buttocks, and around the knee. High-energy trauma can separate the subcutaneous tissue from the underlying deep fascia, causing disruption of perforating capillaries and lymphatic vessels. This creates a potential space that fills with blood, lymphatic fluid, and necrotic fat [1].

These injuries are commonly associated with pelvic or acetabular fractures following major trauma, although isolated lesions may also occur after lower-energy mechanisms [1]. Diagnosis can be challenging because lesions may initially be small and clinically inconspicuous. Progressive accumulation of blood and lymphatic fluid within the degloved tissue plane can result in gradual enlargement over days to weeks, contributing to delayed recognition. Approximately one-third of MLL are diagnosed months or even years after the initial injury [2].

Early recognition is important because management differs between acute and chronic lesions. Acute lesions may respond to compression, aspiration, or minimally invasive drainage, whereas chronic lesions with pseudocapsule formation are more likely to require operative intervention [2]. Failure to recognize the condition may result in progressive enlargement, persistent pain, infection, and increasing treatment complexity.

We present a chronic MLL of the thigh that progressively enlarged over four months following a motorcycle accident. This case highlights the diagnostic challenges associated with delayed recognition and emphasizes the importance of maintaining a high index of suspicion in patients presenting with persistent post-traumatic soft tissue swelling.

## Case presentation

A 35-year-old man was involved in a motorcycle accident after colliding with a car. The left side of his body sustained the majority of the impact, resulting in pain in his left chest and thigh. At the initial hospital, he was diagnosed with a left third rib fracture and a soft-tissue injury of the left thigh. Over the following four months, he experienced persistent pain and progressive swelling of the left thigh, which gradually increased in size from approximately the size of a knuckle. Four months after the injury, he presented to our facility because of persistent swelling and increasing discomfort.

The patient was in the overweight BMI range. On examination, there was a large, tender, warm swelling over the lateral aspect of the left thigh. Hip and knee range of motion were preserved, and he remained independently ambulatory despite the swelling. Laboratory investigations demonstrated leukocytosis (WBC: 21 × 10^9^/L) and an elevated C-reactive protein level of 354 mg/L. Plain radiographs demonstrated soft-tissue swelling over the lateral thigh. Ultrasound demonstrated a large, well-defined, loculated, thin-walled, predominantly anechoic fluid collection, measuring approximately 2.5 × 1.9 × 31 cm between the deep subcutaneous tissue and muscle plane at the anterolateral aspect of the left thigh (Figure 1). CT demonstrated a large multiloculated deep subcutaneous fluid collection extending from the proximal lateral thigh to the distal posterolateral thigh, measuring approximately 12.5 × 5.8 × 29.1 cm, consistent with a Morel-Lavallée lesion (Figure 2).

**Figure 1:**
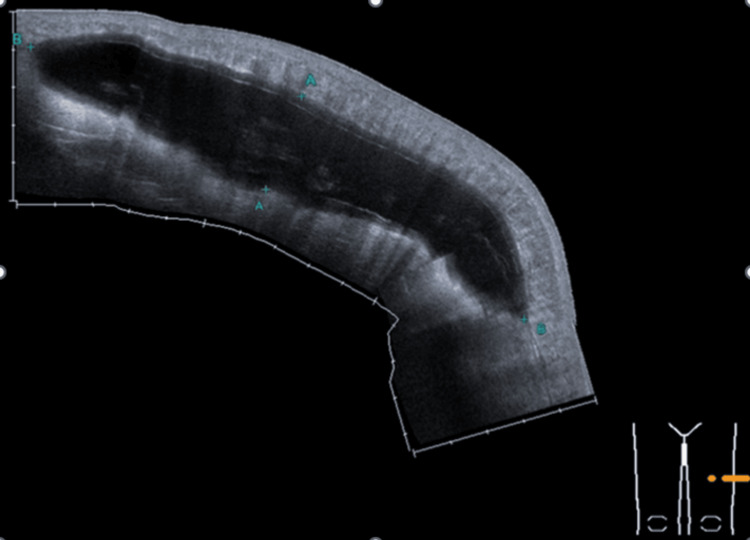
Panoramic ultrasound image demonstrating a large, well-defined, loculated, thin-walled, predominantly anechoic fluid collection with mild internal echogenicity between the deep subcutaneous tissue and the underlying muscle plane at the anterolateral aspect of the thigh, extending toward the posterolateral thigh.

**Figure 2:**
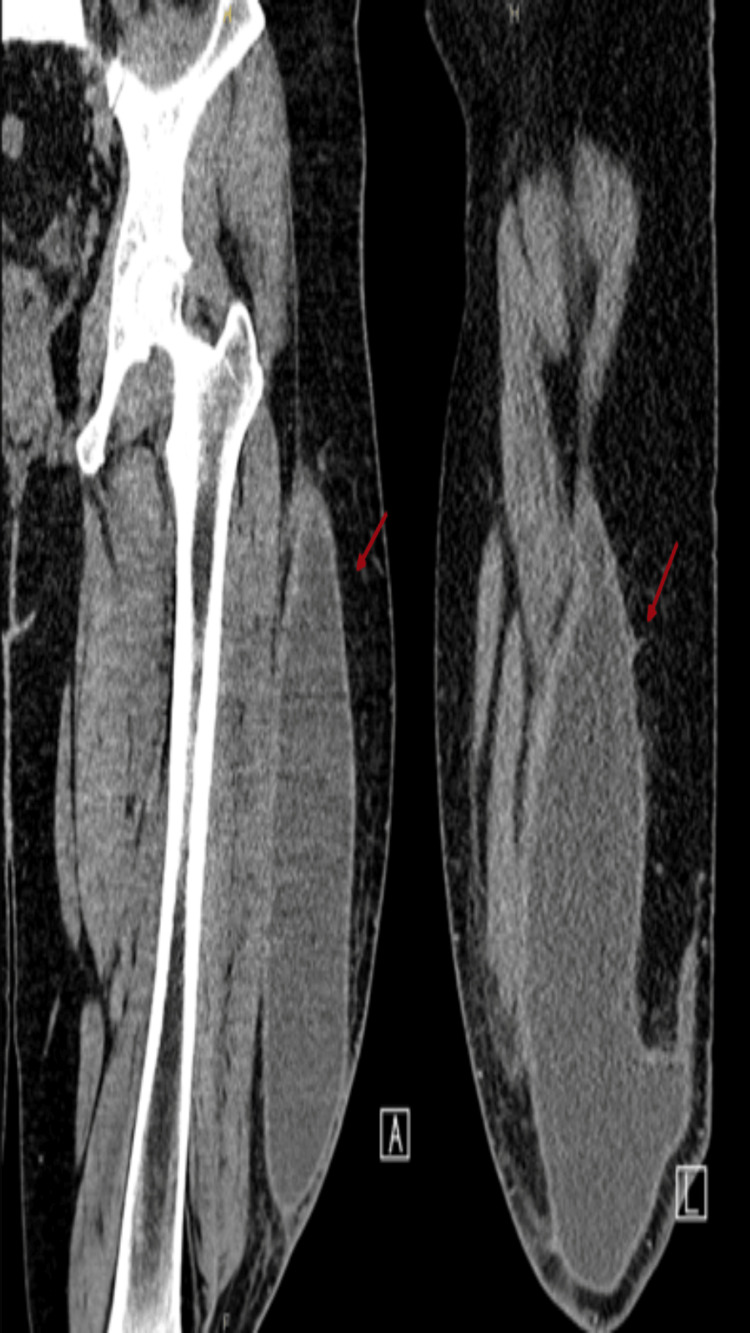
CT scan (left: coronal; right: sagittal) demonstrating a large, multiloculated deep subcutaneous fluid collection within the lateral aspect of the left thigh (red arrows), consistent with a Morel-Lavallée lesion.

Following discussion of treatment options, the patient consented to incision and drainage. Two longitudinal posterolateral thigh incisions were made to facilitate access to both the proximal and distal components of the extensive multiloculated collection (Figure 3). Approximately 1 L of chronic hemoserous fluid was evacuated, and the fascia remained intact without evidence of underlying muscle involvement (Figure 4). Fluid cultures were obtained and subsequently demonstrated no bacterial growth. Empiric antibiotic therapy commenced, and delayed primary closure was performed one week later to allow continued drainage, reduce dead space, and monitor wound progress prior to definitive closure. He remained independently ambulatory throughout admission and was discharged in good condition. At two-week follow-up, the wound was healing satisfactorily without evidence of recurrent fluid collection.

**Figure 3:**
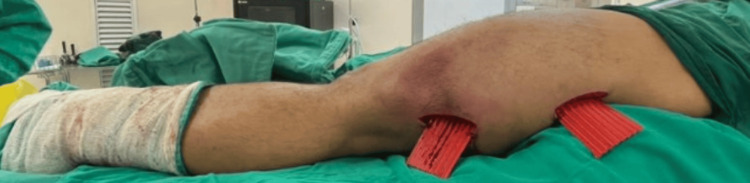
Two longitudinal incisions over the left posterolateral thigh used for surgical drainage of the Morel-Lavallée lesion.

**Figure 4:**
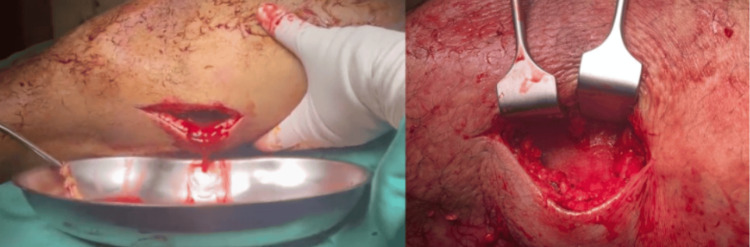
Intraoperative findings. Left: evacuation of chronic hemoserous fluid from the Morel-Lavallée cavity; right: intact fascia of the vastus lateralis, demonstrating preservation of the underlying muscular compartment.

## Discussion

MLL are uncommon soft-tissue injuries that may be difficult to recognize during the acute post-traumatic period. Although they are often described as missed injuries, many lesions are initially small and clinically subtle. Progressive accumulation of blood and lymphatic fluid within the degloved tissue plane can result in gradual enlargement over days to weeks, contributing to delayed recognition [2,3]. These injuries are commonly associated with high-energy trauma involving the pelvis or hip but may occur anywhere that mobile subcutaneous tissue overlies deep fascia [3].

Our patient illustrates this pattern. Following a motorcycle accident, he was initially diagnosed with a rib fracture and a soft-tissue injury of the thigh. The swelling was initially only the size of a knuckle but progressively enlarged over four months before specialist reassessment. Once blood vessels and lymphatics are disrupted, continued fluid accumulation may occur within the potential space created by the degloving injury. Over time, a fibrous pseudocapsule develops, making chronic lesions less likely to resolve spontaneously and increasing the risk of misdiagnosis as abscesses, soft-tissue tumours, or organised haematomas [4].

Early recognition is important because management differs between acute and chronic lesions. Acute lesions may respond to compression therapy, aspiration, or minimally invasive drainage, whereas chronic lesions with established pseudocapsules frequently require operative intervention [5]. In our patient, imaging demonstrated a large multiloculated collection extending along the lateral thigh. The presence of marked inflammatory markers, local inflammatory signs, and the chronic nature of the lesion made conservative treatment unlikely to succeed.

Open surgical drainage was therefore performed, resulting in the evacuation of approximately 1 L of chronic hemoserous fluid. The underlying fascia remained intact, confirming the suprafascial nature of the lesion. Two longitudinal incisions were used to provide access to both the proximal and distal components of the extensive multiloculated collection. Delayed primary closure was performed following a period of wound observation and drainage. At two-week follow-up, the wound was healing satisfactorily with no evidence of recurrent fluid collection.

This case highlights the importance of considering MLL in patients presenting with persistent or progressively enlarging soft-tissue swelling following blunt trauma. When symptoms fail to follow the expected course of a simple soft-tissue injury, further evaluation with ultrasound, CT, or MRI should be considered [4]. Delayed recognition may allow progressive enlargement, pseudocapsule formation, and increasing treatment complexity. Maintaining a broad differential diagnosis remains essential when assessing chronic post-traumatic soft-tissue swellings.

## Conclusions

MLL should be considered in patients presenting with persistent or progressively enlarging soft-tissue swelling following trauma. As demonstrated in this case, these lesions may initially be small and clinically inconspicuous before gradually enlarging over time, contributing to delayed recognition. Once chronic changes and pseudocapsule formation occur, conservative treatment is less likely to be successful, and operative intervention may be required. Appropriate clinical suspicion and timely imaging can facilitate diagnosis and help prevent progression to more complex chronic lesions.
